# Comparison of Subjective and Objective Cognitive Function and Emotional State in Supratentorial Brain Tumors Before Surgery—Recognizing the Influence of Laterality

**DOI:** 10.1002/cam4.70721

**Published:** 2025-02-20

**Authors:** Lisa Schock, Karsten Wrede, Neriman Oezkan, Philipp Dammann, Marvin Darkwah Oppong, Oliver Gembruch, Ramazan Jabbarli, Ilonka Kreitschmann‐Andermahr, Sonja Siegel, Anna Lena Friedel, Adrian Engel, Hanah Hadice Karadachi, Lilith Philomena Laflör, Ulrich Sure, Yahya Ahmadipour

**Affiliations:** ^1^ Department of Neurosurgery and Spine Surgery University Hospital Essen, University of Duisburg‐Essen Essen Germany; ^2^ Center for Translational Neuro‐ & Behavioral Sciences (C‐TNBS) University of Duisburg‐Essen Essen Germany; ^3^ German Cancer Consortium (DKTK) Partner Site University Hospital Essen, University of Duisburg‐Essen Essen Germany; ^4^ Institute for Medical Education University of Duisburg‐Essen Essen Germany

**Keywords:** anxiety, brain tumor, depression, laterality, subjective cognitive function

## Abstract

**Objective:**

Because of its high prognostic value, neuropsychological assessment plays a crucial role in the neuro‐oncology setting. Subjective and objective cognitive performance correlate only to a limited extent, and subjective cognitive performance is strongly dependent on emotional state. We postulate that the relation of subjective and objective cognitive performance depends on tumor laterality.

**Methods:**

In this prospective study, *N* = 63 patients with brain tumors underwent a neuropsychological test battery, including assessment of subjective cognitive function (attention, memory, executive), and symptoms of depression and anxiety before surgery. Patients with psychiatric comorbidity or severe neurological conditions were excluded.

**Results:**

There were no significant differences in subjective and objective cognitive function, symptoms of depression and anxiety between left (*N* = 37) and right (*N* = 26) hemisphere tumors. All measures of subjective cognitive function correlated highly significantly with symptoms of depression and anxiety in left hemisphere tumor patients (all *r* ≥ 0.470). In right hemisphere tumor patients, there was no relation between subjective cognitive function and emotional state. Significant laterality differences for correlations of subjective and objective cognitive function were not found and were not significant within the two groups.

**Conclusions:**

Even when unbiased by symptoms of anxiety and depression, right hemisphere tumor patients show the same discrepancy in subjective and objective cognitive function as left hemisphere tumor patients. This discrepancy may be based on a different mechanism in right hemisphere tumor patients.

## 1 Introduction

Measures of subjective cognitive function are obtained through self‐report measures of the level of functioning in various cognitive domains, whereas objective cognitive function is operationalized through performance on psychometric tests [1].

It is well documented that subjective and objective cognitive functioning are only weakly correlated in patients with a brain tumor [2, 3, 4, 5, 6], meaning that patients cannot adequately assess their level of cognitive performance. Brain tumor patients showed both worse and better subjective cognitive function than controls in previous studies [3, 4]. Different implications arise from these findings, such as questioning the ability of neuropsychological assessment to reflect everyday functioning [5] as well as considering the variability of selection of neuropsychological test batteries and the application of individual tests in clinical practice [7]. Moreover, symptoms of depression and anxiety may be important contributors to bias in self‐assessment of cognitive function [2, 3, 4, 5], for example, psychologically distressed patients report poorer cognitive functioning.

Gehring and colleagues [6] searched for predictors of subjective and objective cognitive functioning in patients with gliomas. They found that objective cognitive deficits were associated with sociodemographic (older age, lower education, male sex) and clinical variables (left hemisphere tumor). Lower subjective cognitive function was more closely related to self‐reported physical (motor) and mental health functioning (mental fatigue, lower mental well‐being, and lower future uncertainty) and female sex; communication deficits were associated with both subjective ratings of cognitive function and attention/executive dysfunction. In another study, neither the type nor the location of the brain tumor, nor subjective complaints or psychological distress were important predictors of significantly poorer performance on neuropsychological tests, but psychological distress was associated with subjective cognitive complaints [5]. Adult brain tumor survivors reported significantly poorer subjective cognitive functioning than controls after accounting for symptoms of anxiety; anxiety levels were associated with poorer subjective cognitive functioning after controlling for physical symptoms and objective global cognitive status [3].

In summary, in most previous studies, subjective cognitive function is associated with psychological distress and mental well‐being in brain tumor patients. The role of laterality in this context is not well understood.

Similarly, both subjective and objective cognitive functions have been associated with depression, anxiety, and fatigue in patients with other types of cancer [8].

The impact of brain tumor laterality on cognitive function is an area that has been extensively studied and still lacks consensus. Previous research shows that left and right hemispheric tumor localization are associated with different cognitive impairments, both in terms of quantity and quality [9, 10]. In addition, patients with right‐hemispheric tumors experienced higher levels of anxiety [10], in contrast to other studies showing that tumor laterality was not a factor influencing health‐related quality of life, symptoms of anxiety, and depression [11, 12]. Left‐sided tumors are often associated with language deficits, but right‐sided tumors yield more inconsistent results or a lower risk of cognitive impairment [13, 14]. Nevertheless, right‐hemisphere tumor patients do not experience less burden [11].

To our knowledge, the relevance of tumor laterality to the relation of subjective and objective cognitive performance has not been considered yet, but may be a further step in exploring hemispheric differences in brain tumor patients.

The aim of the present study is to determine whether subjective and objective cognitive function are differentially aligned in left and right hemisphere tumors. Furthermore, we investigate the influence of symptoms of depression and anxiety on subjective cognitive function in left and right hemisphere tumor patients.

The implications of this study include a better understanding of the clinical burden and self‐reported cognitive impairment in brain tumor patients, as well as improvements in the informative value of neuropsychological assessments when left and right hemisphere tumor patients are considered differently.

## 2 Subjects, Materials and Methods

### 2.1 Subjects

The present work was a prospective, cross‐sectional study performed at the Department of Neurosurgery and Spine Surgery, University Hospital Essen, Germany. Patients with elective admission to the hospital for resection of a lateralized supratentorial neoplasm scheduled for the following day were included in the present analysis. With respect to laterality grouping, only cases without an edema signal on the other hemisphere were implemented in the analysis, evaluated in the T2 resolution images. Images were analyzed by a senior consultant neurosurgeon. In cases where radiotherapy has been previously administered, it has been on the side of the existing lesion. This also applies to previous neurosurgical procedures. Data were collected between October 2023 and September 2024. Clinical data of participating patients were collected from medical records. Exclusion criteria were insufficient command of the German language, psychiatric comorbidity, severe neurological disease or dementia, age over 80 years, and Karnofsky Performance Status < 70, needing assistance [15].

### 2.2 Testing Procedure

A 90‐min neuropsychological test battery was administered, with short breaks as needed by the patient. The order of testing was kept as equal as possible for all participants, with a predefined sequence and the subjective cognitive rating after approximately 45 min of testing. In a few rare cases, the testing had to be done in several stages due to urgent medical examinations. Required intervals between memory tests were filled with other tests. Instructions were given in a standardized manner, taking into account patient queries when necessary. The laterality aspect was reflected by the use of verbal and non‐verbal memory tasks within the same design, respectively, as well as a test for directing attention into both hemi‐fields (visual scanning).

### 2.3 Self‐Report Questionnaires

#### 2.3.1 Depression/Anxiety

Symptoms of depression and anxiety were measured with the Hospital Anxiety and Depression Scale (HADS‐D, [16]) in the German version. It contains 14 statements about the mental state and four possible answers from agreement to disagreement, coded from 0 to 3. Seven items make up the anxiety scale, 7 items make up the depression scale. Scores from 8 to 10 represent elevated values; scores ≥ 11 indicate clinically relevant symptoms.

#### 2.3.2 Subjective Cognitive Symptoms

*The Questionnaire for Cognitive Complaints* (Fragebogen zur geistigen Leistungsfaehigkeit, FLei) is a patient‐reported outcome measure that assesses subjectively experienced mental abilities using a five‐point response scale with 35 questions [17, 18]. Patients report how often in the past 6 months they have experienced problems in everyday life, such as remembering the plot of a book or planning a typical day. Performance in the areas of memory, attention, and executive function is summarized on separate scales; the global score is calculated by summing the three scales. Higher raw values represent worse subjective cognitive function.

### 2.4 Neuropsychological Assessment

#### 2.4.1 Language

##### 2.4.1.1 Boston Naming Test (From CERAD, [19])

The patient is asked to name pictures with one word. The task contains 15 pictures, 5 pictures each with high, medium, and low frequency. A cut‐off score of 13 correct was set for exclusion from the study.

##### 2.4.1.2 Token Test (From Aachener Aphasie Test, AAT, [20])

In 5 runs of 10 items each, the patient is asked to show different combinations of circular or square tiles in five different colors and two different sizes, measuring language comprehension. A cut‐off score of 10 incorrect responses was set for exclusion from the study.

These two tests were not analyzed further since they were only used to rule out aphasia.

#### 2.4.2 Attention

We used two modules from version 2.3.1 of the Test of Attentional Performance (Testbatterie zur Aufmerksamkeitspruefung TAP) ([21]). A measure of general attention (TAP Alertness) and a measure of lateralized attention (TAP Visual Scanning) were included.

##### 2.4.2.1 TAP Alertness

In the intrinsic alertness condition, a cross appears on the monitor at random intervals; the patient must respond as quickly as possible by pressing a key. In the extrinsic alertness condition, the critical stimulus is preceded by a cue stimulus presented as a warning tone. The difference between the intrinsic and extrinsic alertness is the phasic alertness score. A cut‐off percentile of < 1 in the intrinsic alertness condition was set for exclusion from the study. The median reaction time in milliseconds of the no‐sound trial and the phasic alertness score were included in the analysis.

##### 2.4.2.2 TAP Visual Scanning

A matrix of 5 × 5 stimuli is presented, and the patient must determine whether or not this array contains a critical stimulus. Two response keys are used, one for the “present” response and the other for the “absent” response. We performed the analysis with the visual scanning speed: the median response time in trials without a critical stimulus.

#### 2.4.3 Executive Function

Executive function was measured by observing practical activities of daily living (BOPAT, [22]). The test consists of four different tasks: complex sorting (bank statements), arithmetic (addition of outstanding bank transfers, subtraction from account balance), sorting (mail), and structuring (shopping list). Three measures were taken: the quality score for all subtasks combined, the time taken for the subtasks, and a combined score for the speed–accuracy trade‐off, with the score divided by the time in minutes.

#### 2.4.4 Memory

The Figural Memory Test (FGT, [23]) assesses short‐term and long‐term figural memory. In five learning and recall runs, 9 figures are presented repeatedly and must be recalled by mouse clicks after each run. After a five‐minute break, the second part of the test follows with free reproduction of the figures. The third part of the test consists of free recall after a delay of 10–30 min and forced‐choice recognition (familiar or novel figure) of 18 figures.

The Verbal Learning and Memory Test (VLMT) is a test of serial list learning of 15 words [24]. The list is repeated 5 times and must be reproduced after each run. This is followed by a distraction list with immediate recall. After the distraction list, the patient is asked to repeat the first list without hearing the words again. After an interval of 20–30 min, free recall and forced‐choice recognition with semantic and phonemic distractors follow.

For both tasks, the total number of correctly reported items (5 learning trials, short interval recall, long interval recall, recognition) was included in the analysis. For the verbal task, recognition was corrected for errors; for the nonverbal task, false‐positive errors were analyzed separately.

### 2.5 Data Analysis

Data analysis was performed using IBM SPSS Statistics 29 (IBM Corp. Released 2023. IBM SPSS Statistics for Windows, Version 29.0.2.0 Armonk, NY: IBM Corp.).

Descriptive statistics were performed for sample characteristics, cognitive tests, ratings of subjective cognitive function, depression, and anxiety.

Descriptive statistics for cognitive tests and ratings comprised the median, 25th and 75th percentiles for the whole group, because of robustness to potential outliers. Extreme outliers were defined as lying outside the triple interquartile range.

Percentiles relative to age‐ and education‐specific norms were used to categorize performance relative to the general population. The Kolmogorov–Smirnov test for one sample was conducted to test for statistically significant differences in comparison to the normal population.

For all other analyses, the raw values of each test of the test battery described above and the ratings were considered.

Fisher's exact/Freeman–Halton test was used to test for possible differences in sample characteristics between the left and right hemisphere tumor groups with respect to nominal data. Tests for normal distribution led to the decision to perform comparisons for cognitive tests and ratings using nonparametric tests (Mann–Whitney *U* test, Wilcoxon‐*W*, *U*‐ and *Z* values). In addition, the distribution of data between the left and right tumor groups was compared (Kolmogorov–Smirnov‐*Z* for two samples) to meet the requirements for comparability.

Spearman correlation analyses were performed for subjective cognitive measures and symptoms of depression and anxiety, and for subjective and objective cognitive measures. Correlations were compared between left and right tumor groups with Fisher's *Z*‐transformation, one‐sided, because the Spearman correlation coefficients already indicated the direction of the statistical relation.

Bonferroni–Holm correction was applied within each analysis step to adjust for multiple comparisons; the steps are comparisons of cognitive tests and ratings to the normal population (19 tests), left versus right hemisphere tumor group comparisons for cognitive tests and ratings (21 tests), correlation of anxiety and depression ratings with subjective cognitive function ratings (6 tests for the left, the right hemisphere tumor group and for Fisher's *Z*‐transformation each), and correlation of subjective and objective cognitive function (15 tests each).

Statistical significance was set at an alpha level of *p* < 0.050.

## 3 Results

### 3.1 Sample Characteristics

A total of 63 participants (tumor left *N* = 37, tumor right *N* = 26) with complete data sets were included in the analysis. The sample consisted of 36 females and 27 males. The mean age was 52.35 years, with a standard deviation of 14.05 years. Age ranged from 17 to 75 years. Two patients did not undergo tumor resection. At the time of measurement, surgical treatment was scheduled for the following day.

Fisher's exact/Freeman–Halton tests yielded no differences between the left and the right hemisphere tumor groups in the variables of sample characteristics listed in Table 1 (all *p* ≥ 0.208), except for localization. For detailed descriptives, confer to Table 1.

**TABLE 1:** Descriptive characteristic values of the sample, divided by tumor laterality.

Variables	Data	Absolute frequency (*f*) or valid percent tumor left (*N* = 37)	Tumor right (*N* = 26)
Type of tumor	Meningioma	21	10
	Glioma	9	6
	Glioblastoma	3	6
	Metastasis	3	2
	Lymphoma	0	2
	Hemangiopericytoma	1	0
Localization	Frontal	23	7
	Temporal	3	12
	Parietal	9	3
	Occipital	2	2
	Subcortical	0	1
	Ventricular	0	1
WHO grade	1	20	12
	2	4	1
	3	4	1
	4	6	8
	Unclear/not applicable	3	4
Previous brain surgery		16.20%	26.90%
Radiotherapy		10.80%	15.40%
Chemotherapy (past)		13.50%	26.90%
Chemotherapy (current)		2.70%	3.80%
Epilepsy		21.60%	23.10%

*Note:* Absolute frequencies or valid percent of the demographic data of the sample.

### 3.2 Cognitive Function and Symptoms of Depression and Anxiety

**FIGURE 1:**
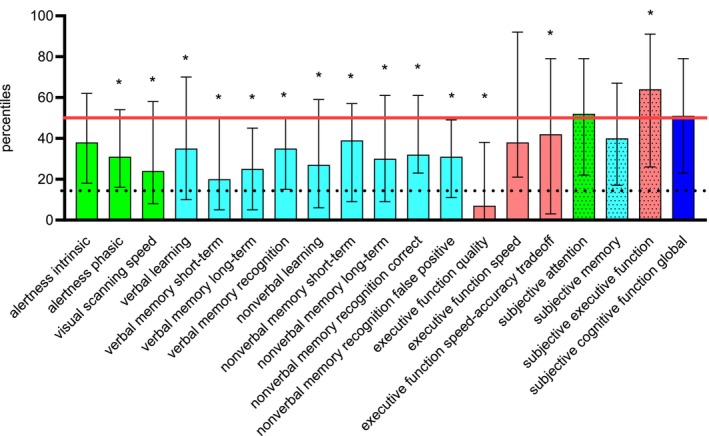
Median, 25th and 75th percentiles (upper and lower limit of error bars) of objective and subjective cognitive domains; higher percentiles indicate better function. The red line indicates the mean of the normal population; the dotted line indicates clinical relevant impairment. The dotted bars are the results of the subjective cognitive measures in the same color as the corresponding objective scores. Green: Attention, light blue: Memory, red: Executive function, dark blue: Global subjective cognitive function. * Significantly different from the normal population, *p* < 0.050.

With respect to the normal healthy population, the cognitive performance in most domains was reduced before surgery on a group level (all Kolmogorov–Smirnov‐*Z* ≥ 1.74, all *p* ≤ 0.030, Bonferroni–Holm corrected), but global subjective cognitive functioning approached the average of the normal healthy population (Kolmogorov–Smirnov‐*Z* = 0.73, *p* > 0.999). The variability throughout the cohort is indicated by the 25th and 75th percentiles; extreme outliers did not occur. Compare Figure 1.

The median depression score was 4.00 (25%ile 2.00; 75%ile 7.00); the median anxiety score was 8.00 (5.00; 11.00) indicating that, on average, there was no clinically relevant depression or anxiety, but anxiety was slightly increased.

### 3.3 Group Comparisons

There were no differences in cognitive parameters, symptoms of depression and anxiety, and subjective cognitive function between the left and right hemisphere tumor groups (all *U* ≥ 269.00, *Z* ≥ ‐3.01 and ≤ ‐0.021, *p* ≥ 0.063, Bonferroni–Holm corrected).

### 3.4 Correlations

Correlations of subjective cognitive function with depression and anxiety scores were highly significant in patients with left hemisphere tumors (all *r* ≥ 0.470, all *p* ≤ 0.006), with higher depression and anxiety scores associated with worse subjective cognitive function (higher raw values FLei). In patients with right hemisphere tumors, there were no significant correlations (all *r* ≤ 0.377, all *p* ≥ 0.342). Group comparisons yielded significance for the correlation of anxiety scores and subjective attention (*Z* = 2.50, *p* = 0.036). Refer to Table 2.

**TABLE 2:** Correlation of subjective cognitive function (FLei) with depression and anxiety scores in left‐and right‐hemisphere tumor patients and group comparisons of correlations.

Variables	FLei attention			FLei memory			FLei executive function		
	Left	Right	Fisher *Z* scores	Left	Right	Fisher *Z* scores	Left	Right	Fisher *Z* scores
HADS‐D depression	0.539**	0.296	1.10	0.557**	0.089	2.00	0.594**	0.377	1.06
HADS‐D anxiety	0.632**	0.069	2.50*	0.470**	−0.088	2.22	0.488**	0.167	1.35

*Note:* Displayed are Spearman correlation coefficients; Fisher Z scores indicate differences of correlations between the left and right hemisphere tumor group.

* Significant at the level of ≤ 0.05 (1‐sided), Bonferroni‐Holm corrected.

** Significant at the level of ≤ 0.01 (2‐sided), Bonferroni‐Holm corrected.

No significant correlations were found for subjective and objective cognitive function (all *p* ≥ 0.585 left hemisphere tumor group, all *p* ≥ 0.960 right hemisphere tumor group, Bonferroni–Holm corrected). No significant differences emerged when statistically comparing correlations between groups (all *p* ≥ 0.375). For detailed results, confer to Tables 3, 4, 5.

**TABLE 3:** Correlation of subjective (FLei) and objective attention function (TAP Alertness, TAP Visual Scanning) in left and right hemisphere tumor patients and group comparisons of correlations.

Variables	FLei attention		
	Left (*r* [95% CI])	Right (*r* [95% CI])	Fisher *Z* scores
Alertness intrinsic	−0.341 [−0.605, −0.009]	0.173 [−0.242, 0.534]^a^	−1.96
Alertness phasic	−0.208 [−0.506, 0.134]^a^	0.111 [−0.299, 0.487]	−1.19
Visual scanning speed	−0.075 [−0.398, 0.265]	0.145 [−0.268, 0.513]^a^	−0.82

*Note:* Displayed are Spearman correlation coefficients with 95% confidence intervals (CI).

^a^ Correlations that indicate concordance of subjective and objective cognitive function regarding the direction of the correlation, for example, a higher FLei attention score and a higher alertness intrinsic score are concordant, because both represent greater impairment, resulting in a positive correlation; Fisher *Z* scores indicate differences of correlations between the left and right hemisphere tumor group.

**TABLE 4:** Memory function (VLMT verbal, FGT nonverbal).

Variables	FLei memory		
	Left (*r* [95% CI])	Right (*r* [95% CI])	Fisher *Z* scores
Verbal learning	−0.328 [−0.596, 0.005]^a^	−0.070 [−0.455, 0.337]^a^	−1.00
Verbal memory short‐term	−0.292 [−0.570, 0.045]^a^	−0.369 [−0.668, 0.034]^a^	0.32
Verbal memory long‐term	−0.306 [−0.580, 0.030]^a^	−0.063 [−0.450, 0.343]^a^	−0.94
Verbal memory recognition	−0.145 [−0.456, 0.198]^a^	−0.140 [−0.509, 0.273]^a^	−0.02
Nonverbal learning	−0.181 [−0.485, 0.162]^a^	−0.150 [−0.517, 0.264]^a^	−0.12
Nonverbal memory short‐term	−0.281 [−0.561, 0.058]^a^	−0.056 [−0.443, 0.350]^a^	−0.86
Nonverbal memory long‐term	−0.140 [−0.451, 0.203]^a^	−0.204 [−0.557, 0.210]^a^	0.24
Nonverbal memory recognition correct	−0.012 [−0.343, 0.322]^a^	−0.329 [−0.643, 0.078]^a^	1.22
Nonverbal memory recognition false positive	−0.114 [−0.430, 0.228]	0.278 [−0.134, 0.608]^a^	−1.48

*Note:* Displayed are Spearman correlation coefficients with 95% confidence intervals (CI).

^a^ Correlations that indicate concordance of subjective and objective cognitive function regarding the direction of the correlation, for example, a higher FLei memory score and a lower verbal learning score are concordant, because both represent greater impairment, resulting in a negative correlation; Fisher *Z* scores indicate differences of correlations between the left and right hemisphere tumor group.

**TABLE 5:** Executive function (BOPAT).

Variables	FLei executive		
	Left (*r* [95% CI])	Right (*r* [95% CI])	Fisher *Z* scores
Executive function quality	0.001 [−0.332, 0.334]	−0.149 [−0.516, 0.264]^a^	0.56
Executive function speed	0.208 [−0.134, 0.506]^a^	−0.134 [−0.505, 0.278]	1.28
Executive function speed‐accuracy tradeoff	−0.140 [−0.452, 0.202]^a^	0.075 [−0.332, 0.459]	−0.80

*Note:* Displayed are Spearman correlation coefficients with 95% confidence intervals (CI).

^a^ Correlations that indicate concordance of subjective and objective cognitive function regarding the direction of the correlation, for example, a higher FLei executive score and a lower executive function quality score are concordant, because both represent greater impairment, resulting in a negative correlation; Fisher *Z* scores indicate differences of correlations between the left and right hemisphere tumor group.

## 4 Discussion

We analyzed the influence of symptoms of depression and anxiety on subjective cognitive function and the relation of subjective and objective cognitive function in brain tumor patients. We focused on the laterality of the tumor as the core of our scientific question, rather than on the location or entity of the tumor. Our findings yielded highly significant correlations of symptoms of depression and anxiety with subjective cognitive function only in left hemisphere tumor patients, but no differences between left and right hemisphere tumor patients in correlations of subjective and objective cognitive function.

The patient group in this present sample was not severely impaired on average, but performed below 50% of the normal population in almost all cognitive domains; the worst performance was obtained in the quality measure of executive function. Subjective cognitive function was close to the mean of the normal population. Van Lonkhuizen et al. found impairments in attention and cognitive flexibility before surgery in a group of meningioma patients without significant changes three and 12 months after surgery [4]. Patients with different tumor entities showed significantly worse cognitive performance than healthy controls before treatment [10, 25]. The present results are consistent with previous studies showing that cognitive performance is reduced in brain tumor patients before treatment compared to the normal population.

In the present sample, there were no significant differences in cognitive parameters, symptoms of depression and anxiety, and subjective cognitive function between left‐and right‐hemisphere tumors. Neuropsychological impairment differed between left‐ and right‐sided glioblastomas, leading to delayed diagnosis due to minor clinical abnormalities [26]: Aphasia is a core symptom of left‐sided tumors, whereas attention deficits are more common in right‐sided tumors. However, it has been shown previously that there are different laterality patterns in attention and verbal tasks in different tumor entities [9, 10, 27, 28]. In the present study, we explicitly excluded moderate‐to‐severe aphasia and severe alertness deficits to account for these differences and to avoid confounding with other measured functions.

We found that subjective cognitive function was correlated with anxiety and depression, as in many previous studies [2, 3, 4, 5, 6]. Anxiety was related to subjective cognitive function in different tumor entities and at different time points of assessment [3, 4]. In our cohort, left hemisphere tumor patients showed a fairly strong association of symptoms of depression and anxiety with subjective cognitive function, whereas right hemisphere tumor patients did not show this association. The FLei questionnaire was developed for psychiatric patients [18] and may therefore measure subjective cognitive complaints differently than similar instruments. That is, the FLei asks about general, not specific, experiences [17] and does not differentiate between the constructs of perceived cognitive impairment and perceived cognitive ability [3]. Since right hemisphere impairment is associated with poorer self‐reflection of cognitive function [29], here, assessment of subjective cognitive function may be biased by worse self‐reflection, including worse ability to reflect on general experiences, rather than biased by symptoms of depression and anxiety.

In line with previous studies, the associations of subjective and objective cognitive function remain low and non‐significant [4, 6]. Additionally, we did not find significant group differences in the correlations of subjective and objective cognitive function. Left hemisphere tumor patients show the same discrepancy of subjective and objective cognitive function as right hemisphere tumor patients, but are biased by symptoms of depression and anxiety. The results, therefore, point to hemispheric differences in the underlying mechanism of this discordance, like cognitive awareness, independently of cognitive status and self‐reported cognitive function, for example, metacognition. Metacognition is defined as an aspect of information processing and monitoring of processes, whereas cognitive awareness serves as a broader concept of self‐perception: Patients with right hemisphere high‐grade glioma showed worse cognitive awareness, even with similar neurocognitive status [30]. Another concept for the differences in awareness of functioning in left and right hemisphere damage involves a less conscious right hemisphere mode of operation [31]. This model explains anosognosia in patients with right hemisphere damage by a less conscious and more automatic functioning of the right hemisphere in contrast to the highly intentional, language‐structured functioning of the left hemisphere.

The main finding of the present study is that tumor laterality may distinguish between the effect of depression/anxiety on subjective cognitive function and other mechanisms influencing the relation of subjective and objective cognitive function in brain tumor patients.

Regarding the clinical implications of the present results, the effectiveness of cognitive rehabilitation after brain tumor surgery may depend on the specific cognitive domains affected by tumor location and laterality, but also on the patient's ability to reflect on these deficits. Baseline cognitive status is a consistent predictor of (long‐term) outcomes [13, 32, 33]. Learning more about the predictors of cognitive outcomes after surgery, such as the role of subjective cognitive function and the role of tumor laterality in these outcomes, may help tailor rehabilitation and treatment plans to individual patient needs.

The strength of the present study is that even with a small sample size, the effect sizes are moderate to high, as compared to similar studies [4, 6]. Nevertheless, the external validity of the results would benefit from future research with larger sample sizes and postoperative evaluation to corroborate and extend the present findings. Another strength of the present study is that we recruited patients in a single center over a 12‐month period with a highly standardized test battery and consistent preoperative institutional conditions. We consistently corrected for multiple comparisons and therefore report only robust statistical relations. We excluded patients with a history of neurological or mental illness.

Limitations of the present study include the heterogeneity of the sample. On the other hand, laterality, which was the key factor in the current study, did not show statistical differences in sample characteristics, depression, anxiety, subjective cognitive function, and all cognitive parameters. Tumor localization was different, with more frontal tumors in the left and more temporal tumors in the right hemisphere. It has previously been shown that patient‐reported cognitive function in brain tumor patients is associated with the left hemisphere, but not necessarily with frontal regions [34], so that the effect may not be based on tumor location. In addition, we controlled for comorbidities, aphasia, and severe alertness deficits to reduce heterogeneity. Within the groups, there are different tumor entities that may experience different cognitive burdens. However, our goal was to include as many tumor entities as possible to show that the influence of laterality is pivotal. Furthermore, we accounted for this fact by including only patients with a Karnofsky Performance Status ≥ 70, for example, patients who were not severely clinically impaired [15]. Previous studies also included different tumor entities and tumor sizes, as well as treatment backgrounds [5, 6, 10]. Tumor type, for instance, was not associated with specific neurocognitive impairments [10, 35]. Tumor location showed differential influence [5, 10]. A history of chemo‐ and/or radiotherapy did not predict cognitive function [6].

## 5 Conclusion and Outlook

Left and right hemisphere tumor patients differ in the relation of depression and anxiety symptoms to subjective cognitive function but not in the relation of subjective and objective cognitive function. We suggest that reduced awareness leads to worse self‐reflection in right hemisphere tumor patients, whereas in left hemisphere tumor patients, symptoms of anxiety and depression may bias the evaluation of their own cognitive status.

The current literature suggests that there is a wide variation in cognitive performance and self‐reported cognitive function in brain tumor patients, with most patients showing cognitive impairment prior to treatment but not being able to adequately assess their performance—in line with the present findings. In the field of neuropsychological diagnostics, there is an urgent need to determine with which tests and under what circumstances subjective and objective cognitive performance are best matched. On the current state of research, a comprehensive neuropsychological assessment is essential, as self‐assessment remains inadequate.

## Author Contributions

Conceptualization: Lisa Schock, Yahya Ahmadipour. Methodology: Lisa Schock, Yahya Ahmadipour. Formal analysis and investigation: Lisa Schock, Lilith Philomena Laflör. Writing – original draft preparation: Lisa Schock, Yahya Ahmadipour, Ilonka Kreitschmann‐Andermahr, Lilith Philomena Laflör, Anna Lena Friedel, Sonja Siegel. Writing – review and editing: Lisa Schock, Karsten Wrede, Neriman Oezkan, Philipp Dammann, Marvin Darkwah Oppong, Oliver Gembruch, Ramazan Jabbarli, Ilonka Kreitschmann‐Andermahr, Sonja Siegel, Anna Lena Friedel, Adrian Engel, Hanah Hadice Karadachi, Lilith Philomena Laflör, Ulrich Sure, Yahya Ahmadipour. Funding acquisition: Lisa Schock; Resources: Yahya Ahmadipour, Ilonka Kreitschmann‐Andermahr, Ulrich Sure. Supervision: Yahya Ahmadipour, Ilonka Kreitschmann‐Andermahr.

## Ethics Statement

The study was approved by the local ethics committee of the University Hospital Essen (23‐11434‐BO) and was conducted according to the tenets of the Declaration of Helsinki.

## Consent

All patients gave written informed consent before participation in the study.

## Conflicts of Interest

The authors declare no conflicts of interest.

## Data Availability

Any data not published within the article will be shared in an anonymized manner at the request of any qualified investigator.
